# Who is calling? Optimizing source identification from marmoset vocalizations with hierarchical machine learning classifiers

**DOI:** 10.1098/rsif.2023.0399

**Published:** 2023-10-18

**Authors:** Nikhil Phaniraj, Kaja Wierucka, Yvonne Zürcher, Judith M. Burkart

**Affiliations:** ^1^ Institute of Evolutionary Anthropology (IEA), University of Zurich, Winterthurerstrasse 190, 8057 Zürich, Switzerland; ^2^ Neuroscience Center Zurich (ZNZ), University of Zurich and ETH Zurich, Winterthurerstrasse 190, 8057 Zürich, Switzerland; ^3^ Department of Biology, Indian Institute of Science Education and Research (IISER) Pune, Dr. Homi Bhabha Road, Pune 411008, India; ^4^ Behavioral Ecology & Sociobiology Unit, German Primate Center, Leibniz Institute for Primate Research, Kellnerweg 4, 37077 Göttingen, Germany; ^5^ Center for the Interdisciplinary Study of Language Evolution (ISLE), University of Zurich, Affolternstrasse 56, 8050 Zürich, Switzerland

**Keywords:** machine learning, hierarchical classifier, marmoset calls, bioacoustics, time series analysis, source identification

## Abstract

With their highly social nature and complex vocal communication system, marmosets are important models for comparative studies of vocal communication and, eventually, language evolution. However, our knowledge about marmoset vocalizations predominantly originates from playback studies or vocal interactions between dyads, and there is a need to move towards studying group-level communication dynamics. Efficient source identification from marmoset vocalizations is essential for this challenge, and machine learning algorithms (MLAs) can aid it. Here we built a pipeline capable of plentiful feature extraction, meaningful feature selection, and supervised classification of vocalizations of up to 18 marmosets. We optimized the classifier by building a hierarchical MLA that first learned to determine the sex of the source, narrowed down the possible source individuals based on their sex and then determined the source identity. We were able to correctly identify the source individual with high precisions (87.21%–94.42%, depending on call type, and up to 97.79% after the removal of twins from the dataset). We also examine the robustness of identification across varying sample sizes. Our pipeline is a promising tool not only for source identification from marmoset vocalizations but also for analysing vocalizations of other species.

## 1. Introduction

Comparative studies of primate communication are crucial for understanding the evolutionary origins of human speech [1–4]. Much progress has been achieved over the last decades, mainly by recording and simultaneously annotating the vocalizer or physically separating social partners to track individual contributions to conversations. The vocal communication of callitrichid monkeys appears particularly rich among non-human primates, with several features thought to be precursors of language. For instance, common marmosets (*Callithrix jacchus*) have been shown to possess superior control and flexibility in their calls, one of the requirements for speech. Among others, they can actively interrupt ongoing calls [5,6], make long-term changes to call frequency in response to noise [5], converge in vocal space to a social partner [7] and engage in antiphonal call conversations similar to antiphonal speech in humans [8]. As immatures, they go through a babbling phase [9], and contingent vocal feedback from caregivers contributes to fully developing their repertoire [10,11], not described in any other primate and reminiscent of vocal learning—an essential building block for language. Marmosets may be particularly relevant because, like humans, they are cooperative breeders, which may have played a major role in language evolution [12]. However, our knowledge of how marmosets and other primates communicate under more naturalistic situations and beyond dyadic contexts is rather limited (but see [13]). Given the highly social nature of marmosets, the next frontier in studying marmoset vocal communication is to do so under more naturalistic conditions—in social groups, where they fully display their communication skills.

A major bottleneck for studying group-level communication in naturalistic settings without separating animals is to accurately determine the vocalizer (source identity). There are at least two approaches to doing this. On the one hand, microphone arrays can localize sounds in three-dimensional space. This information can then be integrated with visual monitoring or signal-based individual localization to match the vocalization to its source individual. This is mostly feasible in captive conditions. On the other hand, a more broadly applicable alternative is to use individual signatures in the vocalizations to classify calls.

Previous attempts to determine source identity from animal vocalizations have used a broad range of unsupervised (e.g. k-means [14], Gaussian mixture models [14], Bayesian functional mixed models [15], hidden Markov models [16]) and supervised machine learning (ML) classifiers (e.g. discriminant function analyses [17], support vector machines [18], random forests [19], artificial neural networks [20]) mostly with mixed results (16.26–91.5% accuracy, but see [21,22] for instances of greater than 95% accuracy). To improve classifier performance, the focus has largely been on hyperparameter optimization [23], feature selection [24] and data quality enhancement techniques [25]. However, for instances of supervised classification, training datasets available to researchers often contain not only individual identities but additional information such as the sex, age class or social status of the individual. Such additional information could aid in improving classifier performance when implemented in hierarchical machine learning algorithms (MLAs). Furthermore, increasing the number of features extracted before feature selection would give the classifier a more detailed representation of the vocalizations and a larger feature space. The current study aims to demonstrate this for source identification from marmoset vocalizations by addressing the two issues with (i) plentiful feature extraction to achieve detailed representations of the calls and (ii) hierarchical MLAs that take sex into account as the first hierarchical layer.

First, the traditional approach to representing animal vocalizations involves spectral feature extraction. Specific spectral features are chosen because we understand how sound modifications affect these feature values, making them easy to interpret. Software like Raven (The Cornell Lab of Ornithology, Ithaca, NY, USA), Avisoft SASLab Pro (Avisoft Bioacoustics, Germany) and Kaleidoscope (Wildlife Acoustics Inc., USA) offer easy solutions to extracting these features and have been widely used along with custom scripts for feature extraction from animal vocalizations. A drawback of this approach is that not all animal vocalizations show maximum variability along these ‘pre-selected’ feature values. The small feature space and reduced class separability of calls due to limited features prevent ML approaches from achieving their maximum potential for obtaining high classification accuracies. One workaround is to initially extract a vast number of acoustic features and let the MLAs decide which ones would be most helpful for their classification task. For this, recent advances in time series analyses allow for multiple operations to be performed that provide meaningful information about the nature of the time series [26]. This can be exploited by viewing the acoustic waveform as a time series of pressure points and performing time-series analyses on acoustic data. In contrast to spectrogram-based feature extraction software, time series analyses can extract up to 7700 features from a single vocalization [27]. In this paper, we implement time-series analysis for plentiful feature extraction and use tree-based classifiers to extract the most meaningful features. This enables us to provide detailed representations of the marmoset calls as input for the classifier.

**Figure 1.**
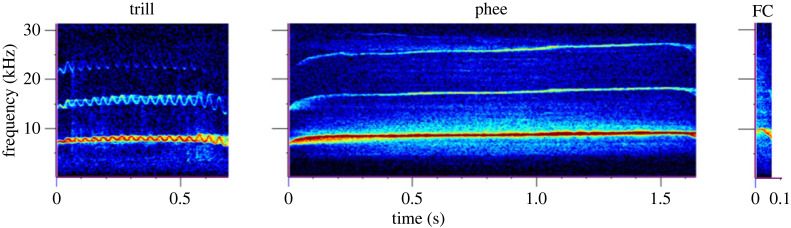
Marmoset call types. Example spectrograms of a single trill, phee and food call (FC, 0.08s in length) of common marmosets. Spectrograms were obtained using the Hann filter of size 512 samples, hop length of 256 samples and discrete Fourier transform (DFT) bin size of 512 samples.

Second, we hypothesize that using a hierarchical ML classifier will improve classification accuracies by breaking up the classification problem into a hierarchy of smaller ones. Callitrichid vocalizations contain information about the sex of the caller [28–30], which can potentially assist MLAs with efficient source identification. We, therefore, develop a hierarchical ML classifier that first determines the sex of the source, thus narrowing down the possible source individuals, and then determines the source identity. We compare its performance with the non-hierarchical approach by applying it to three major marmoset call types relevant to group coordination and affiliative behaviours: trills, phees and food calls. Trills are used as within-group close-contact signals. They are between 0.3 and 0.8 s in duration and are characterized by quick sinusoidal variations in pitch between 5 and 8 kHz throughout the call [31]. Phees are louder long-distance contact calls with variable durations between 0.5 and 2 s and a narrow frequency band between 6 and 8 kHz [31]. Food calls are extremely short (approx. 0.05 s), steep downsweeps (10–6 kHz) elicited by individuals when they see food, during feeding and to initiate food sharing [31,32]. Example spectrograms for each call type are provided in figure 1. Finally, we analyse the acoustic features used by the hierarchical classifier for the various steps in the hierarchy and compare the precisions and recalls of the non-hierarchical and hierarchical classifiers at different sample sizes (i.e. number of calls per individual).

## 2. Methods

### 2.1. Experimental subjects

This study used marmoset vocalizations collected by Zürcher *et al*. [7]. The data contained vocalizations from 20 adult common marmosets, 10 males and 10 females. They were housed in 2.4 × 1.8 × 3.6 m enclosures with at least one other individual, with each group having access to a personal outdoor enclosure of the same dimension and a common experimental room. Lighting was regulated to maintain a 12/12 h day/night cycle. Animals were fed a predetermined amount of vitamin-enriched mush in the morning, vegetables and fruits during noon, and one of either gum, boiled egg, cottage cheese or insects after noon. Ad libitum access to water was always provided. All experiments were approved by Zürich's cantonal veterinary office (licence ZH223/16).

### 2.2. Vocalization recordings and segmentation

Individuals were recorded in their home enclosures or in a separate experimental room in sessions lasting for approximately 30 min. Each individual went through approximately 17 recording sessions (17.3 ± 15.5 mean ± s.d.) spread over approximately 8 days (7.6 ± 7.6 mean ± s.d.). As phee calls are long-distance contact calls, focal individuals were separated from their group to elicit them. In this case, the focal individual was only visually isolated but acoustically in contact with other group members. For eliciting food calls, the focal individual's highly preferred food was provided until enough vocalizations were obtained during that session. Trills could be recorded without any such interference. A condenser microphone (CM16/CMPA, Avisoft Bioacoustics, Germany) connected to Avisoft UltraSoundGate 116H (Avisoft Bioacoustics, Germany) was used for recordings, and calls were labelled in real time using Avisoft Recorder (Avisoft Bioacoustics, Germany). Calls were recorded at a sampling rate of 62 500 Hz and segmented manually using Avisoft Pro (Avisoft Bioacoustics, Germany). The start and end of calls were determined by visual inspection of the spectrogram. Only those calls that were visible clearly on the spectrogram, had no interference with other calls and could be accurately assigned to the categories of trill, phee or food call were included for further analyses. See [7,33] for detailed information about the recording procedure and processing.

### 2.3. Datasets and feature extraction

An imbalanced dataset with a skewed data distribution biases the classifier towards the majority class, often preventing it from learning the underlying patterns that make the classes different and limiting its generalizability. Such a problem can be solved by balancing the dataset. As the original dataset was imbalanced (the number of calls per call type per individual was highly variable), a combination of majority class random undersampling and synthetic minority oversampling technique (SMOTE) [34,35] was used to create 18 datasets (one original and five generated, for each of the three call types). SMOTE is a data augmentation technique that synthesizes new data for minority classes without repeating the original datapoints to make them equal to the majority class. For this, first, a call belonging to a minority class was randomly selected (observation). Next, the nearest minority class ‘neighbours’ of this call in the high-dimensional feature space (and not necessarily belonging to the same individual) were determined. Then, the feature vector of the observation was subtracted from that of the nearest neighbours, multiplied by a random number between 0 and 1, and added to the feature vector of the observation. This effectively synthesized new data points within the hypervolume bounded by the neighbours. The datasets are listed as follows (X = T for trills, P for phees, F for food calls in the name of the dataset):

1. Original-X: The original dataset after feature extraction. Consisted of 1247 trills, 1443 phees and 4434 food calls from 20 individuals each.
2. Imbalanced-X: Marmosets with less than 25 calls per call type were removed from the Original-X datasets to make them suitable for ML processing. No undersampling was done. Consisted of 1207 trills from 16 individuals, 1374 phees from 10 individuals and 4419 food calls from 18 individuals.
3. Balanced-X: SMOTE was applied on Imbalanced-X to obtain balanced datasets. Consisted of 4528 trills, 3350 phees and 15 372 food calls.
4. Balanced197-X: From the Imbalanced-X dataset, classes with greater than 197 calls were undersampled to 197 calls. SMOTE was applied to this. Consisted of 3152 trills, 1970 phees and 3546 food calls.
5. Balanced99-X: From the Imbalanced-X dataset, classes with greater than 99 calls were undersampled to 99 calls. SMOTE was applied to this. Consisted of 1584 trills, 990 phees and 1782 food calls.
6. Balanced50-X: From the Imbalanced-X dataset, classes with greater than 50 calls were undersampled to 50 calls. SMOTE was applied to this. Consisted of 800 trills, 500 phees and 900 food calls.

The smaller Balanced197-X, Balanced99-X and Balanced50-X datasets were used to test the capability of the ML approach to classify calls in limited sample size scenarios.

For feature extraction, the MATLAB-based highly comparative time series analysis (HCTSA) [27] toolbox provides an architecture to extract over 7700 features from every call, and we implemented this for marmoset calls. When inputted, HCTSA views the acoustic waveform as a time series of pressure points, performs several time-series analyses on acoustic data, and provides a matrix of feature measurements. Features common across calls of all individuals for a given call type were used for further analyses. As every call is a point in a very high-dimensional feature space, we required a dimensional reduction technique to visualize the trill, phee and food call datasets. We did so using t-distributed stochastic neighbour embedding (t-SNE) [36], an unsupervised MLA for nonlinear dimensional reduction.

We trained individual multi-class adaptive boosting algorithms with decision trees as weak learners and 10-fold cross-validation (henceforth AdaBoost) for determining the important features to use for classification and for performing the classification itself. This method uses multiple weak learners to create a strong learner [37]. Unlike random forests, in which multiple trees are trained in parallel, and their collective decision (using a ‘voting’ system) is obtained, AdaBoost trains trees in a sequential manner wherein every new tree aims to be specialized in correcting the errors of a previous tree. AdaBoost is considered better than random forest classifiers due to its higher accuracy and lower susceptibility to overfitting [38,39]. We first trained AdaBoost (using MATLAB's ‘fitcensemble’, ‘AdaboostM1′, and ‘AdaboostM2′ functions) on Imbalanced-X, Balanced-X, Balanced197-X, and Balanced99-X datasets to classify calls based on source identity—as a direct or ‘non-hierarchical approach’ (in contrast to the hierarchical approach that was used later)—to determine source identity from calls. We chose the number of trees and learning rate based on the observations of the classification loss function.

Although classification accuracy is the most widely used metric to assess MLAs, it does not represent the model's performance on class-imbalanced datasets. This is because the classifier can get away with a high accuracy score by simply predicting most data points as belonging to the majority class. In such cases, the receiver operating characteristic (ROC) curve can be used to evaluate the classifier's performance. The ROC curve visualizes how the true positive rate changes as a function of the false positive rate at various threshold values. The area under this ROC curve, simply ‘area under curve’ (AUC), can be a useful tool for examining classifier performance along with accuracy. Therefore, for assessing the performance of AdaBoost on Imbalanced-X datasets, ROC-AUC was calculated in a one versus rest setting for each class in the dataset, along with the accuracies.

Even while assessing the performance of MLAs on balanced datasets, accuracy does not represent how variable the predictions for each class (marmoset individuals) are. Class-specific precision and recall values represent individual-specific performance of the classifier and how they vary across individuals. Therefore, for the rest of the classifiers, precisions and recalls for each class were calculated using the formulae below, and the summary (means and standard deviations) of these values was used to assess their performance.

$$\mathrm{precision}=\frac{\mathrm{true}\;\mathrm{positives}}{\mathrm{true}\;\mathrm{positives}+\mathrm{false}\;\mathrm{positives}}$$

and

$$\mathrm{recall}=\frac{\mathrm{true}\;\mathrm{positives}}{\mathrm{true}\;\mathrm{positives}+\mathrm{false}\;\mathrm{negatives}}.$$

**Figure 2.**
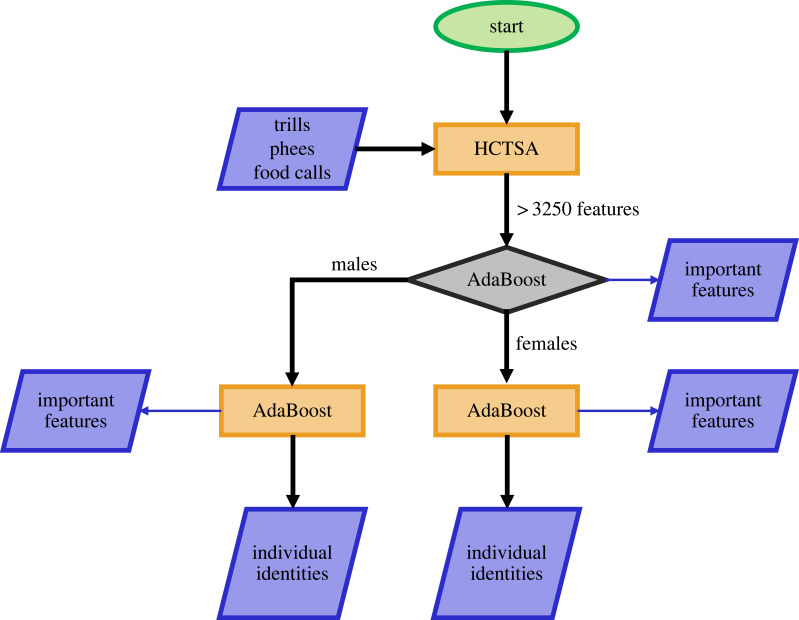
The hierarchical classification approach. Features from trills, phees, and food calls were extracted using HCTSA. These features were used to train AdaBoost to first classify calls based on sex and then individual identities. The oval denotes start, parallelograms inputs/outputs, rectangles processes, and the diamond a decision.

For inspecting if individual variability of calls within marmoset groups can be explained by variation in sex and whether MLAs could exploit this to perform better, individual AdaBoosts were trained on Imbalanced-X and Balanced-X datasets to classify calls based on sex, and then the source individual. Sex was chosen as a cue because previous studies in callitrichids have shown that they can discriminate calls based on the sex of the source [28–30], and in our case, the total number of classes could be split into half based on the sex of the individual (10 males and 10 females out of 20 individuals). Later, each dataset was divided into two sub-datasets based on the ‘true’ sex of the source individual, and separate AdaBoosts were trained on each of them. This was the hierarchical approach to determine first the sex and then the source identity from calls (figure 2). To assess the performance of the classifiers for the hierarchical classification approach, precisions and recalls for each individual were calculated for all classifiers as

$$Y_{\;}^{\mathrm{final}}(\mathrm{sex},\mathrm{ind})=\;Y(\mathrm{sex})\times Y(\mathrm{ind}\;|\;\mathrm{sex}).$$

*Y* is the precision or recall, and ind is the individual identity of the marmoset. For example, the final precision for a female individual would be the precision for determining the sex as a female, multiplied by the precision for determining the source individual among females.

We performed Wilcoxon signed-rank test to compare precision and recall scores for every class between the two approaches because the classes, i.e. the individuals for both approaches, were the same.

### 2.4. Feature importance scoring

Along with classifying calls, each fold of AdaBoost also provides predictor importance scores for each feature, which represents how important that feature was for AdaBoost in the classification task. First, we checked how (non-hierarchical) AdaBoost performed the feature selection task. For this, we ranked features by predictor importance scores given by the most accurate of the 10 models (run on 10 different folds) in the AdaBoost for each dataset. Then, for the three Balanced-X datasets, we used the top-20 features to visualize t-SNE clusters. t-SNE plots were generated using MATLAB's ‘tsne’ function with the Barnes–Hut algorithm keeping the Barnes–Hut trade-off parameter at 0.5 to increase processing speed for large datasets. Exaggeration was set to 4, perplexity to *n*/100, and learning rate to *n*/12 (where *n* is the total number of calls for that call type) as these values are shown to provide robust results when datasets are large [40]. We compared these with t-SNE plots of the corresponding datasets generated using 20 random features. For a more quantitative comparison, we selected 20 random features from each of the three datasets 100 times, plotted the histograms of the mean silhouette scores, fit Gaussians to these distributions, and calculated the probability of getting a mean silhouette score greater than that of the top-20 features by chance. For the hierarchical classifiers, we analysed the features used by the various levels in the hierarchy when implemented on the three Balanced-X datasets.

## 3. Results

### 3.1. Datasets and feature extraction

The sample sizes of each of the datasets obtained are listed in table 1. The Original-X and Imbalanced-X datasets have variable number of calls per call type and individual (electronic supplementary material, table S1).

**Table 1.** Sample sizes of the datasets.

dataset	trills			phees			food calls		
	female	male	total	female	male	total	female	male	total
Original-X	895	352	1247	565	878	1443	1906	2528	4434
Imbalanced-X	895	312	1207	533	841	1374	1904	2515	4419
Balanced-X	2547	1981	4528	1420	1930	3350	7686	7686	15 372
Balanced197-X	1773	1379	3152	788	1182	1970	1773	1773	3546
Balanced99-X	891	693	1584	396	594	990	891	891	1782
Balanced50-X	450	350	800	200	300	500	450	450	900

We could extract between 3776 and 4553 features from each marmoset call in the Original-X datasets, with 3255, 3395 and 3477 features common across the calls of all individuals for trills, phees and food calls, respectively.

### 3.2. Machine learning classifiers and feature importance scoring

We monitored the classification loss function of AdaBoost with the addition of every new weak learner. We observed the loss function to plateau at approximately 500 trees while training AdaBoost to determine sex and approximately 2500 for other tasks, and these number of trees were therefore used.

#### 3.2.1. Balanced versus unbalanced datasets

Classification accuracies ranged from 60.8% to 70.96% for imbalanced datasets versus 71.43% to 83.92% for balanced datasets (chance probabilities = 6.25% for trills, 10% for phees and 5.56% for food calls). Mean ROC-AUCs were higher for all the Balanced-X datasets compared with Imbalanced-X datasets (table 2).

**Table 2.** Non-hierarchical AdaBoost performance for imbalanced and balanced datasets. Imbalanced-X and Balanced-X datasets were used. Mean ± s.d. are provided for ROC-AUC scores.

call	classes (individuals)	imbalanced			balanced		
		sample size	ROC-AUC (%)	accuracy (%)	sample size	ROC-AUC (%)	accuracy (%)
trills	16	1206	92.63 ± 4.30	64.84	4528	98.95 ± 0.91	82.91
phees	10	1498	94.36 ± 4.54	70.96	3550	98.55 ± 1.42	83.92
food calls	18	4419	92.72 ± 5.08	60.8	15 372	97.04 ± 2.31	71.43

#### 3.2.2. Top-20 versus random-20 features

**Figure 3.**
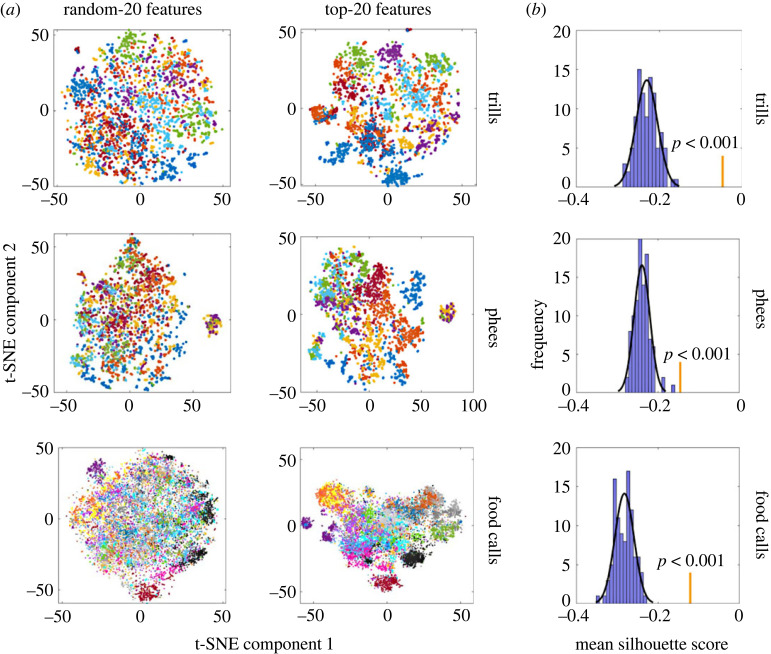
Qualitative and quantitative comparisons of random-20 and top-20 features for clustering data. (*a*) Qualitative comparison. Figures are t-SNE plots (squared Euclidean distance metric) of Balanced-X datasets using 20 randomly chosen features or the top-20 features for classification by AdaBoost for that call type. Each point is a call, coloured according to the source individual. (*b*) Quantitative comparison. Blue bars depict frequencies (histogram) of mean silhouette scores obtained after performing t-SNE using 20 random features selected 100 times for that call type (trills/phees/food calls) on Balanced-X datasets. The grey line depicts the Gaussian function fit to the histogram. Orange vertical bars denote the mean silhouette scores obtained after performing t-SNE using top-20 features for classification by AdaBoost for that call type. The *p*-value shown is the normalized area under the Gaussian function to the right of the orange bar.

t-SNE plots obtained using top-20 features on the Balanced-X datasets showed visibly better clusters compared with t-SNE plots obtained using random-20 features on the corresponding datasets (figure 3, electronic supplementary material, tables S2–S4). The top-20 features provided by AdaBoost gave significantly greater silhouette scores than what would be obtained by chance for trills (*Z* = 7.0154, *p* < 0.001), phees (*Z* = 4.8361, *p* < 0.001) and food calls (*Z* = 6.8922, *p* < 0.001).

#### 3.2.3. Hierarchical versus non-hierarchical classifiers at large sample sizes

**Figure 4.**
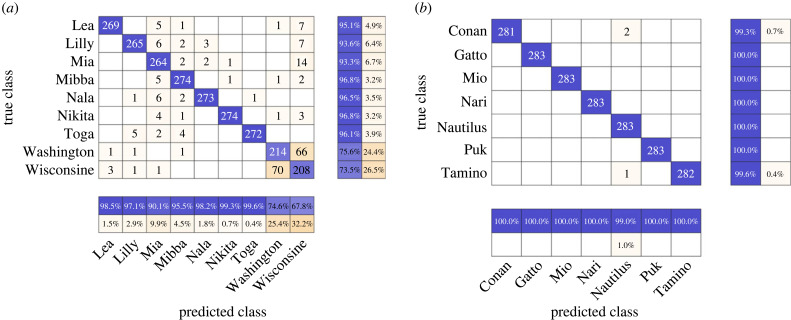
Individual precisions and recalls for determining the source identity from trills by the hierarchical classifier for females (*a*) and males (*b*). Confusion matrices are shown, with rows depicting the true source identity and columns representing the prediction made by the hierarchical classifier. The absolute number of calls is shown within the matrices, with those correctly classified highlighted in blue and those wrongly classified highlighted in orange (intensity proportional to the number for both). The rows and columns are summarized with the row summary depicting individual precisions in blue and the columns summary showing individual recalls in blue. The classifiers were tested on the Balanced-T datasets.

The accuracies of AdaBoost to classify the following datasets based on sex were: Balanced-T = 99.4%, Balanced-*p* = 96.8%, Balanced-F = 96.7%. The hierarchical classification approach gave significantly better precision and recall scores than the non-hierarchical approach for the corresponding Balanced-X datasets (*p* < 0.05 across all call types, Wilcoxon signed-rank test; table 3). A thorough evaluation of the precisions and recalls for each individual by the hierarchical classifier revealed that the trills of two individuals—Washington and Wisconsine (female twins)—had significantly lower scores (precisions: 74.23% and 67.46%, respectively, recalls: 75.07% and 72.99%, figure 4). The mean precision and recalls for trills for all except these two individuals were as high as 97.79% and 97.07%, respectively. The phee precision and recall scores for Washington and Wisconsine were also slightly lower than that of other females (electronic supplementary material, figures S1*a,b*). For food calls, the precision and recall scores of these two individuals were similar to or higher than the mean scores of all females (electronic supplementary material, figure S1*c,d*).

**Table 3.** Comparing non-hierarchical and hierarchical approaches for classifying calls based on source identity. Mean ± s.d. precisions and recalls with corresponding *p*-values for testing the hypotheses: mean precisions/recalls of non-hierarchical = hierarchical. Both approaches were tested using the Balanced-X datasets.

call	classes (individuals)	non-hierarchical		hierarchical		*p*-value	
		precision (%)	recall (%)	precision (%)	recall (%)	for precision	for recall
trills	16	83.42 ± 11.48	82.87 ± 8.63	94.42 ± 9.61	94.19 ± 8.24	0.017	0.01
phees	10	84.15 ± 9.84	83.93 ± 8.63	92.57 ± 3.30	92.55 ± 3.79	0.027	0.037
food calls	18	72.23 ± 15.09	71.43 ± 13.13	87.21 ± 9.71	86.65 ± 5.62	0.01	0.002

#### 3.2.4. Hierarchical versus non-hierarchical classifiers at reduced sample sizes

**Figure 5.**
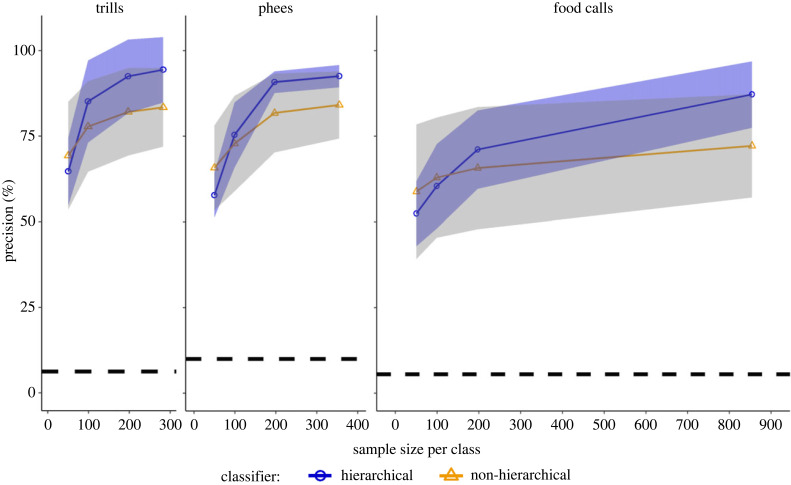
Classifier performance at different sample sizes. Precisions of AdaBoost as a function of sample size per class for trills, phees and food calls with s.d. represented as shaded regions around lines connecting means. Dashed black lines indicate the chance precision of classification for a given call type. Note that the highest sample size per class was obtained by oversampling the minority classes to be equal to the majority class (SMOTE, see Methods). See electronic supplementary material, table S1 for sample sizes in our original dataset.

With the decrease in sample size per class, the difference between the mean precisions and recalls of the hierarchical and non-hierarchical approaches was reduced for all three call types (figure 5 for precision, electronic supplementary material, figure S3 for recall).

#### 3.2.5. Feature selection at various levels of the hierarchical classifier

**Figure 6.**
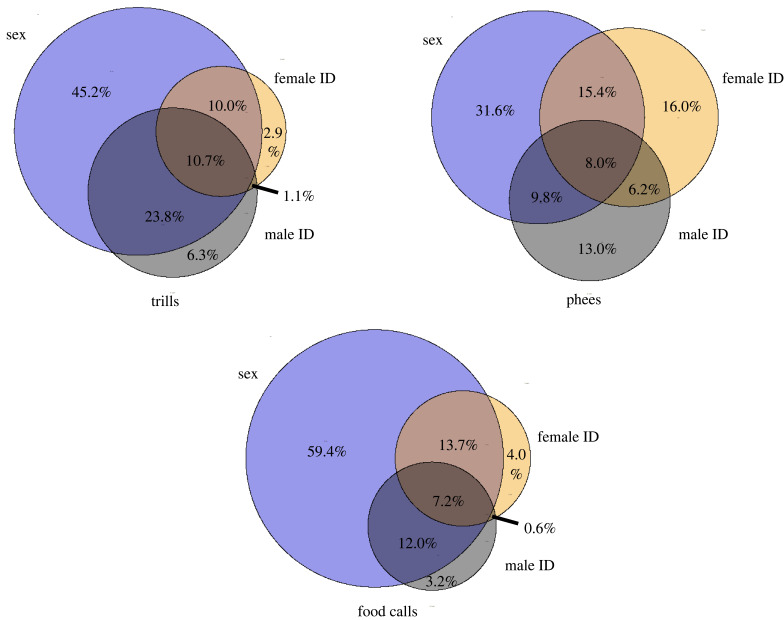
Mutual and distinct features used by hierarchical classifiers at various levels of classification. Venn diagrams denote the set of features used for determining the sex, source identity among females (female ID), and source identity among males (male ID). The area of the circle is scaled to the number of features at that level. The percentage of total features used by the hierarchical classifier belonging to each area within the Venn diagram is denoted.

The features selected by each level of the hierarchy varied with the task. Approximately 30%–60% of the features were used solely for determining sex across call types. Only a few features were common for determining individual identities among males and females (figure 6). The family of features from which most of the top-10 important features for each classification task came are presented in electronic supplementary material, table S5.

## 4. Discussion

We show that the information about sex and source identity encoded in marmoset calls can be harnessed for constructing hierarchical ML classifiers. Such hierarchical classifiers exhibit higher precisions and recalls than their non-hierarchical counterparts. Our findings have implications for marmoset communication research and will benefit understanding group-level communication dynamics. The same methods can also be extended for efficient source identification in other species.

### 4.1. Optimizing source identification

Of the large number of features extracted with HCTSA, we found that the AdaBoost reliably selected the most important features that would best cluster the data for its classification task (figure 3, electronic supplementary material, tables S1–S3). Intriguingly, the selected features varied with the task of the classifier (figure 6). Secker *et al*. [41] have previously used a hierarchical classifier with independent feature selection by the components to classify proteins successfully. They suggest that independent feature selection maintains high predictive performance while improving computational efficiency. In our case, the independent feature selection enabled the hierarchical classifier to efficiently use broader-category cues (i.e. sex) for classification, boosting performance over its non-hierarchical counterpart. AdaBoost thus provides task-specific and flexible feature extraction with a customized set of features for every dataset and can be applied to a wide range of animal vocalization datasets.

Even with the substantial number of features provided by HCTSA, AdaBoost performed poorly on the unbalanced dataset, with accuracies below 70% (table 2). A large body of ML literature points out the problem of class-unbalanced datasets and its solutions [42,43]. Recent reviews have emphasized using data augmentation and balancing techniques to improve ML accuracy when handling acoustic data [44,45]. Consistent with other studies [46,47], balancing the datasets significantly improved the performance of AdaBoost across call types. Therefore, data balancing using tools like SMOTE combined with random undersampling is important before running MLAs on any dataset.

Classifying data points from a noisy dataset to multiple classes (10–18 individuals in our case) is often demanding for an MLA. Here, we broke the problem of classifying calls to over 10 sources into the problem of first assigning the sex of the source and, only then, given the sex, classifying source identity in a second step (figure 2). With this hierarchical approach, we showed that at large sample sizes, mean precisions and recalls across datasets increased by more than eight percentage points (table 3). We found the accuracies of the hierarchical classifier on the largest balanced datasets to remain satisfactory and higher than most recent studies classifying animal vocalizations using MLAs [48–50], despite the number of samples per class of data being lower than those studies. The same was reflected in the performance of the hierarchical classifier on the originally collected calls (electronic supplementary material, figure S2).

The difference in precisions and recalls between the hierarchical and non-hierarchical approaches diminished as the sample sizes decreased, suggesting that the hierarchical classifier requires exposure to enough data to perform significantly better than its non-hierarchical counterpart (figure 5, electronic supplementary material, figure S3). Lower sample sizes pose a higher risk of error cascading in the hierarchical classifier, and this has been identified in similar classifiers developed for text classification [51,52]. However, this limitation only arises at the level of the training dataset.

The requirement of a large training dataset and the presence of a small collected dataset can almost always be bridged. The median sample sizes per individual in our Imbalanced-X datasets were 47 for trills, 83 for phees and 173 for food calls (electronic supplementary material, table S1). Using this small, highly imbalanced dataset, we could generate larger balanced datasets and train and test our classifiers on them (see Methods). Therefore, to obtain optimal performance from the hierarchical classifier, one need not necessarily acquire a large number of calls to begin with (see performance of the hierarchical classifier on the Imbalanced-X dataset in electronic supplementary material, figure S2).

While we find that source identification from vocalizations can be optimized by using broader-category cues with the help of hierarchical classifiers for marmoset calls, the same methods can be extended to other tree-based classifiers and vocalizations from other animals due to two reasons. First, as tree-based classifiers inherently follow a hierarchical decision-making process, we predict that embedding them in a larger hierarchical framework and providing more information about the vocalizations will improve their performance. Second, the ability to select customized features for every dataset and task, combined with the supervised nature of learning, makes our pipeline highly flexible and extendable for analysing vocalizations of diverse animal species. However, because our pipeline uses a supervised MLA, a major requirement is that the calls of all individuals need to be represented in the training dataset. The source identity options available for the classifier to return as the output for any source determination task is simply the set of all the individuals it has encountered in the training dataset. Our method will not be useful, for example, for estimating the number of individuals in a large number of animal vocal recordings, as this information is required as input for the pipeline to work. In such cases, unsupervised MLAs such as Gaussian mixture models and hidden Markov models (see [53,54]) are suitable alternatives. However, the pipeline can classify calls of all individuals it has ‘seen’ during the training step. In the future, we hope to extend the idea to larger, more complex animal vocalization datasets with a greater number of individuals that would require multiple levels in the hierarchy and use other cues, such as the age and social status of the animal, for efficient source identification.

### 4.2. Implications for understanding the marmoset communication system

The classification precisions and recalls for food calls were lower than trills and phees, despite over three times higher sample sizes (table 3). In particular, food calls required a higher sample size per class to be classified as accurately as trills and phees (figure 5). This could be due to three possible reasons, or a combination of them: (i) food calls are a highly heterogeneous group of call types [55]. As the calls function in signalling other individuals about the availability of food and in inducing food sharing, it is highly likely that their acoustic structure is influenced by the food type, the internal state of the marmoset, its motivation to share food, and the social bond strength between the signaller and the surrounding individuals. Our method may therefore show limited performance in cases where the acoustic structure of calls change significantly with time (e.g. through seasons or during development) or context. This can be especially problematic if the variation in calls due to the aforementioned reasons surpass the levels due to inter-individual variability. Nevertheless, a prudent approach would involve uniformly sampling calls across various temporal and contextual settings for training the classifier. (ii) As the primary purpose of food calls is to alert other individuals about a food source, it may be under lower selection pressure to encode source identity information as compared with contact calls like trills and phees. (iii) Food calls are predominantly produced in bouts of multiple repeated call units [56]. Furthermore, each call unit is much shorter than a trill or a phee [56,57], providing reduced information for time series analysis. The poor classification results could thus arise because some of the source identity information may well be encoded at the level of the bout. This is testable in the future by repeating the classification procedure on bouts of food calls.

Intriguingly, the mean precision and recall for determining source identity from trills when considering all except two individuals were as high as 98.38% and 97.65%, respectively (figure 3). The two marmosets with low scores happened to be twins of the same sex. Thus, the low classification scores were probably due to the high vocal similarity between the twins' calls, which is also probably why the female identity classifier performed poorer than the male identity classifier (figure 4). It is known that in multi-class classifiers, decision boundaries for classes are not independent; therefore, poor performance on one class may negatively affect the performance of other classes [58]. Similar patterns were present to a lesser extent for phee calls but not for food calls (electronic supplementary material, figure S1). Whereas the food calls may require further scrutiny at different levels of analyses (see above), the contrast between trills and phees is interpretable with regard to their biological function. Signalling identity is essential for phee calls that individuals typically use to establish acoustic contact when visual contact is not possible [59]. In contrast, trill calls are given in close proximity to a social partner, and the caller's identity is thus redundant (marmosets may also use visual or olfactory cues to identify the partner) [60]. It is, therefore, possible that the twins actively diverged from each other in their phee calls but not in their trill calls. This is consistent with a recent study [33] on newly paired marmosets that found that partners would converge in the structure of their phee calls. However, newly formed pairs that had initially similar phee calls diverged rather than converged in their call structure, supposedly to make themselves better distinguishable.

Callitrichids can differentiate between contact calls originating from cage-mates versus foreign individuals and from males versus females [29,30]. An intriguing question is how they achieve that and whether their decision-making process may likewise be hierarchically structured with broader-category cues used as a first distinction. Multiple studies on humans allude to the hierarchical nature of decision-making in various contexts [61–65]. Some frog species seem to employ hierarchical decision-making for prey capture [66]; a hierarchical decision-making model appears to explain best the strategies used by rhesus macaques while playing a slightly modified, semi-controlled adaptation of a video game [67]; sea lions use a hierarchy of multimodal cues during mother–offspring recognition [68,69]; and evidence from fruit flies, locusts and zebrafish suggest that they break down a complex problem of deciding between spatially distributed options into a series of smaller problems [70]. Hierarchical decision-making may thus be a widespread feature of cognitive processing. Given that sex could be attributed with extremely high precision by our classifier, we hypothesize that marmosets, too, use these sex-based cues for efficient source identification from calls, and they are doing so in a hierarchical manner, similar to the hierarchical classifier. Cognitive and psychological experiments will be required to test this hypothesis.

Even though recent studies have turned towards collecting large amounts of group-level acoustic data from animals [71–74], they lack information about the identity of the callers, which is important for understanding the ecology and behaviour of the species. With few manually labelled vocalizations, one can train our algorithm to determine source identities of a larger number of unlabelled vocalizations. This can be done by passing the unlabelled vocalizations through feature extraction and the same hierarchy of classifiers the labelled dataset was passed through. In this case, the sex labels provided by the first level of the hierarchical classifier are to be used to split the data into male and female vocalizations. These have to be passed through separate individual-level classifiers trained on the vocalizations of the respective sex. Our pipeline is thus an easy yet powerful tool to add source identity information to non-individualized datasets. It will support the analysis of vocal signals from groups of animals simultaneously, in contrast to the traditional method of focusing on one focal individual in a group or isolating individuals for recordings. Such a step is essential for understanding group-level communication dynamics of highly social species like marmosets and shedding light onto the communicative interactions that help in group-level coordination for raising young, which is thought to be a driver for the evolution of language.

## Data Availability

All datasets and codes used in the study can be accessed from the Zenodo repository: https://doi.org/10.5281/zenodo.8367132 [75]. Supplementary figures and tables are provided in electronic supplementary material [76].
